# Violence and Asthma: A Review

**DOI:** 10.4137/EHI.S884

**Published:** 2008-07-25

**Authors:** Takeo Fujiwara

**Affiliations:** Department of Health Promotion and Research, National Institute of Public Health, Wako-shi, Saitama, Japan.

**Keywords:** violence, asthma, stress

## Abstract

Recent research shows that exposure to community violence is, directly and indirectly, associated with asthma. This article reviews the findings on the impact of violence on asthma, and the pathways for the association of violence and asthma are suggested: 1) exposure to violence is directly associated with asthma, mainly through dysregulation of sympathetic-adrenal-medullary (SAM) and hypothalamic-pituitary-adrenal (HPA) axis, 2) exposure to violence is associated with the change of susceptibility of outdoor air pollution on asthma, probably through the change of an immune response, and 3) behavioral change due to exposure to violence (e.g. keeping children indoors) leads to more exposure to indoor pollutants. The suggested framework may be useful to develop health policy on asthma in high-violence communities.

## Introduction

**Figure 1:**
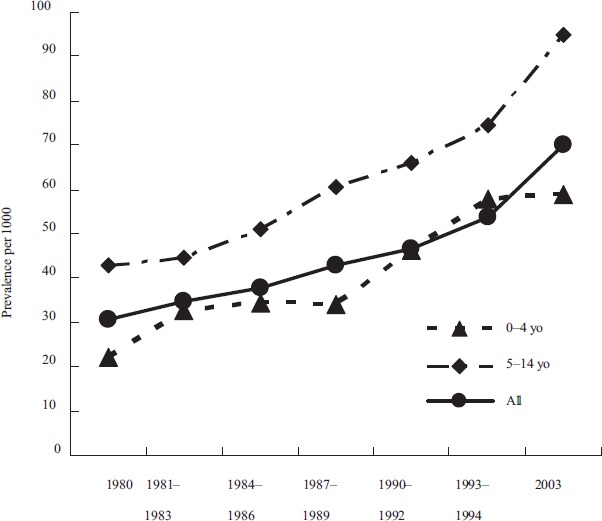
Trend of current asthma prevalence per 1000 from 1980 to 2003. (Source: National Health Interview Survey, CDC).

Current trends indicate that the prevalence rates for current asthma have increased more than double from 1980 to 2003 (Vollmer et al. 1998; Moorman et al. 2007) (Fig. 1). The most substantial increase occurred among children ages 0 to 4 years and ages 5 to 14 years (Moorman et al. 2007). The medical services used to treat asthma result in over 10.8 million physician visits, over 478,000 hospitalizations, 2 million emergency room visits, and about 28 million missed school days annually (Mannino et al. 2002). Direct health care expenditures such as physician visits, medications and other interventions are estimated to be U.S.$7.4 billion (Weiss and Sullivan, 2001).

Research indicates that there is a disproportionate burden of asthma amongst children of low socioeconomic position (Akinbami and Schoendorf, 2002; Gold and Wright, 2005; Litonjua et al. 1999; Claudio et al. 2006; Juhn et al. 2005; Halfon and Newacheck, 1993; Mielck et al. 1996). Living in disadvantaged communities would increase the exposure to outdoor air pollution, such as traffic related air pollution (Graves et al. 1988; Brulle and Pellow, 2006; Maantay, 2007; Evans and Kantrowitz, 2002), and community violence (Selner-O'Hagan et al. 1998; Finkelhor and Dziuba-Leatherman, 1994; Schubiner et al. 1993), and the psychological distress (Stronks et al. 1998). Increased life stress along with low socioeconomic position would be another pathway leading to an effect on asthma (Wright et al. 1998a; Lehrer et al. 1993; Isenberg et al. 1992; Chen et al. 2003), as chronic stress is associated with exacerbation (Oh et al. 2004; Sandberg et al. 2004) and onset (Wright et al. 2002; Klinnert et al. 2001) of asthma. Research in the U.S. showed that the exposure to violence had a direct impact on the development and exacerbation of asthma (Wright and Steinbach, 2001; Wright et al. 2004; Swahn and Bossarte, 2006; Berz et al. 2007), although a recent study in Puerto Rico showed that neither exposure nor victimization of community violence were associated with asthma (Cohen et al. 2008).

The psychosocial stress due to violence might influence asthma through sympathetic-adrenal-medullary (SAM) axis and hypothalamic-pituitary-adrenocortical (HPA) dysregulation, which is associated with inflammation and suppression of immune function. In addition, it is also reported that there may be an interaction effect with air pollution to exacerbate asthma due to exposure to violence: impact of air pollution on asthma is more harmful among those exposed to violence (Clougherty et al. 2007; Chen et al. 2008). Furthermore, exposure to violence would lead to changes in behavior, which might be associated with asthma. For example, due to the fear of community violence, children might be skipping medications, or kept indoors which would increase the exposure to indoor air pollution (Wright et al. 2004; Gold and Wright, 2005). A framework on the association between violence and asthma would be useful for public health practitioners to help develop better measurements for the prevention of asthma, and to prioritize future research on this topic. Thus, the purpose of this study is to review the association between violence and asthma and to describe the possible pathways for the association between exposure to violence and asthma.

## Suggested Pathways between Violence and Asthma

**Figure 2:**
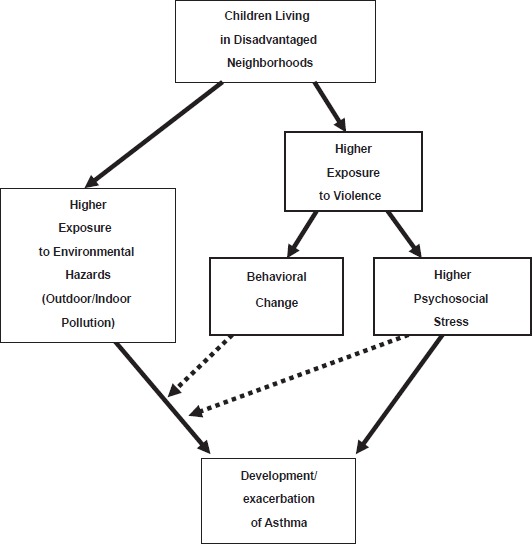
Proposed pathways between violence and asthma.

The suggested pathways between violence and asthma were developed based on previous reviews: Cohen and Herber (1996) and O'Neil et al. (2003). Cohen and Herber showed that psychological characteristics affect immune change through central nervous system innervations, hormonal response, and behavioral change. Then, the immune change influences onset and progression of immune system-mediated disease, including asthma. A theoretical framework of the pathway is social cognitive theory (Bandura, 1982; Bandura, 1989). If appraised environmental demands are higher than perceived adaptive capacity (i.e. low self-efficacy), people considered the environmental demands as stressful, which results in negative emotions such as fear, anger, anxiety, and depression (Cohen et al. 1995). These negative emotions lead to physiological and behavioral responses, which increase the risk of disease (Herbert and Cohen, 1993), including asthma (Ritz et al. 2000). Moreover, even tiny stimuli can have long term effects because of recurrent memories about past events (Baum et al. 1993). Thus, particular negative emotional experiences, such as exposure to violence, may have reinforced pulmonary physiological responses, such as broncoconstriction. In addition, O'Neil et al. suggested that low socioeconomic position may directly increase susceptibility to air pollution-related health consequence, including asthma. Thus, the impact of exposure to violence might affect the susceptibility of air pollution on asthma. Based on this theory, proposed pathways for how violence influences asthma is summarized in Figure 2, including psychosocial stress pathway and behavioral change pathway.

## Psychosocial Stress Pathway of Violence on Asthma

**Figure 3:**
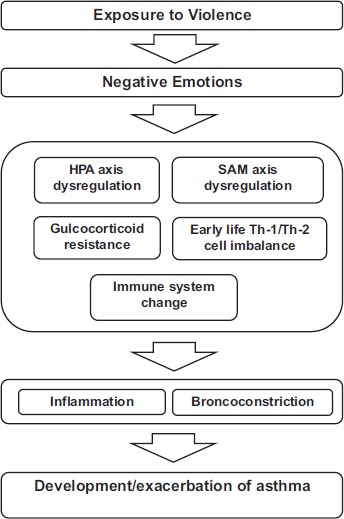
Proposed biological pathways between violence and asthma.

Exposure to violence, especially, witnessing or victimization of violence, is considered to be a type of psychological stress (Breslau et al. 1991; Lehrer et al. 1993; Isenberg et al. 1992; Busse et al. 1995; Gold and Wright, 2005). Psychological stress can be associated with development or exacerbation of asthma as adaptation of allostatic load (McEwen, 1998). The detailed biological pathway of psychological stress is shown in Figure 3. However, perception of violence might differ by individual (Sampson et al. 1997; Curry et al. 2008). Thus, for more precise description, psychological stress due to exposure to violence needs to be linked to negative emotion (Kubzansky and Kawachi, 2000), such as fear or anger. Previous research showed that exposure to violence is associated with adverse psychological consequences among children (Martinez and Richters, 1993; Boney-McCoy and Finkelhor, 1995).

Negative emotion seems to have an effect on asthma, biologically, through four possible pathways: 1) SAM axis dysregulation (Glaser and Kiecolt-Glaser, 2005), 2) HPA axis dysregulation (Ockenfels et al. 1995), 3) gulcocorticoid resistance (Miller et al. 2002), 4) early life Th1/Th2 cell imbalance (Calcagni and Elenkov, 2006), and 5) immune system change (Wright et al. 1998a). These pathways interrelate with each other, and result in inflammation and air flow obstruction.

1. SAM axis dysregulation
  Negative emotion is associated with the activation of SAM axis (Cannon, 1914). Activation releases adrenaline (epinephrine) from adrenal medulla and noradrenaline (norepinephrine) from sympathetic nerve endings. Generally, these stress hormones produce cognitive arousal, sensory vigilance, tachycardia, raised blood pressure, and dronchodilation. However, recent research showed that adrenergic nerves may influence cholinergic neurotransmission in parasympathetic system via prejunctional α and β receptors, which leads broncoconstriction (Barnes, 1995). The relative strength of sympathetic vs. parasympathetic control in response to certain form of stress differs with the individual. Those who have a predominant parasympathetic response system may be particularly susceptible to stress induced broncoconstriction (Lehrer et al. 1993). Studies examining exposure to stress-ors have looked at the parasympathetic system using vagal reactivity to measure stress related to emotion induced airway constriction (Lehrer et al. 1993). The mechanism involves the presence of an acute stress event which will trigger a parasympathetic response including vagal activation and a corresponding rapid release of catecholamine leading to airway constriction.
  In addition, chronic exposure of catecholamine (i.e. adrenaline and noradrenalin) alters immune function (Glaser and Kiecolt-Glaser, 2005), which might contribute to inflammation of the airway. Moreover, chronic stress, which induces prolonged catecholamine level, may lead to the down regulation of β receptors, as chronic daily users of beta agonist showed (Drazen et al. 1996).
2. HPA axis dysregulation
  HPA axis is less rapid response to stressful situation, resulting in cortisol release (Selye, 1936). The hypothalamus produces corticotrophin releasing hormone (CRH) which triggers the anterior pituitary gland to secrete adrenocorticotorophic hormone (ACTH), which intern activates the adrenal cortex to secrete corticosteroids, or cortisol in human. HPA axis can be activated by cytokines, such as TNF-α, IL-1, or IL-6, which are associated with stress (Fukata et al. 1994; Chrousos, 1995). The several feedback loops regulate the activity of the HPA axis. For example, under chronic and stressful situations, cortisol provides feedback to the hypothalamus and the hippocampus in order to regulate further release of cortisol. As a result, the sensitivity to release cortisol declines. Thus, chronic stress induce a state of hypo-responsiveness of the HPA axis whereby cortisol secretion is attenuated, leading to inflammation. Moreover, prolonged increase of cortisol induces immune suppression (Glaser and Kiecolt-Glaser, 2005), preferentially suppression of Th1 function (Chrousos, 1995) and promotes Th2 function (Ramirez et al. 1996), which is associated with asthma.
  More recent evidence suggests that the HPA axis habituates quickly to stress, and that cortisol levels can sometimes rebound below normal (Heim et al. 2000). For example, high levels of work-related burnout are associated with lower levels of cortisol in the morning (Pruessner et al. 1999), and post-traumatic stress disorder is associated with lower cortisol level (Yehuda, 1997). Cortisol in turn has an inhibitory effect on the immune system, signaling immune cells to stop the inflammatory process. Thus, decreased cortisol level is thought to be associated with overactivity of immune system (Chrousos, 1995), leading to increased airway responsiveness and airflow obstruction (Buske-Kirschbaum et al. 2003; Wamboldt et al. 2003; Ball et al. 2006). Studies have shown an association between decreased levels of endogenous cortisol and asthma (Kauffmann et al. 1999; Landstra et al. 2002).
3. Glucocorticoid resistance
  Although chronic stress leads to HPA axis dysfunction, cortisol itself is act as anti-inflammatory hormone. However, recent research showed that chronic psychological stress induce inflammation through glucocorticoid resistance among white blood cells, which produce cytokine related to anti-inflammation (Miller et al. 2002). That is, with continued exposure to high concentration of stress hormones, white blood cells begin a counter-regulatory response and down-regulate the expression of glucocorticoid hormones receptors. Miller et al. (2002) showed that among parents of cancer children, dexamethasone's capacity to suppress the production of IL-6, a pro-inflammatory cytokine, was significantly reduced compared with parents of medically healthy children.
4. Early life Th1/Th2 cell balance
  In conjunction with hygiene hypothesis (Strachan, 1989; von Mutius, 2000), it is argued that early life Th1/Th2 balance is associated with the development of asthma. In a germ-free environment, such as no siblings, use of antibiotics, vaccination, low lactobacillus, and industrialized society, Th2 response is predominant (Umetsu et al. 2002; Weiss, 2002). And Th2 release cytokines such as IL4 or IL5, which induce IgE or eosinophile production (Chung and Barnes, 1999; Barnes, 1994; Marshall and Agarwal, 2000). Thus, Th2 mechanism is considered to be associated with allergic reaction, including asthma (Robinson et al. 1992). Polarization of immune function into an atopic phe-notype, such as Th2 predominant, likely occurs during early childhood and even before birth (Yabuhara et al. 1997; Finn et al. 2000).
  Recent evidence indicates that stress hormones (i.e. cortisol and catecholamine) selectively inhibit the Th1 but potentiate Th2 cytokine production, systematically (Elenkov and Chrousos, 1999; Calcagni and Elenkov, 2006). For example, periods of stress, such as examinations performed by medical students, are associated with a shift toward increased stimulated Th2 cytokine (IL-10) production away from Th1 cytokine (IFN-γ) production (Marshall et al. 1998). Another study also showed that IL-5 (releasing from Th2) and eosinophil were increased under stressful situation (i.e. school examination), and these cytokines were associated with airway inflammation among students with mild allergic asthma (Liu et al. 2002). Thus, stress might induce Th2 shift in Th1/Th2 balance, which is associated with asthma. Although few study exactly assess the association between exposure to violence and Th1/Th2 balance, there is evidence that parental report of life stress is associated with onset of wheezing in children under one year of age (Wright et al. 1996).
5. Immune system change
  Stress can modulate the immune response through autonomic nerves and immune system, by triggering the stress hormone and neuropeptides that interact with immune cells (Cohen and Herbert, 1996). Moreover, the interaction between central nerve system and immune function is more dynamic. Cytokines induced by stress hormones influence the production of CRH by the hypothalamus (Glaser and Kiecolt-Glaser, 2005). Furthermore, nerve fibers in the spleen and thymus provide evidence of direct connections between the SAM and lymphoid organs (Bellinger et al. 2001). Thus, it is better to consider that brain and immune system are communicating (Maier, 2003). Further research on how inflammatory-cytokine network shape mood or cognitive would contribute to elaboration of the mechanism of stress, immune function, and inflammation.

## Indirect Pathway of Violence on Asthma: Interaction

In addition to direct pathways of psychosocial stress on asthma, a recent review suggested that psycho-social stress might change the vulnerability which modifies the effect of toxicants on biological systems (Gee and Payne-Sturges, 2004; O'Neill et al. 2003; Weiss and Bellinger, 2006). It is proposed that low socioeconomic position may directly increase susceptibility to air pollution-related health consequence (O'Neill et al. 2003; Morello-Frosch and Shenassa, 2006). For example, exposures to nitrogen dioxide were found to be significantly and positively associated with asthma hospitalization for males in the low socioeconomic group but not in the high socioeconomic group (Lin et al. 2004).

In the same context, exposure to violence might act as mediator to change the susceptibility of air pollution which is associated with asthma (Clougherty et al. 2007; Chen et al. 2008; Clougherty et al. 2006). Some evidence suggests that stress may influence the internal dose of a given toxicant. This is because stress may increase the absorption of toxicants into the body through increased respiration, perspiration, and consumption (Gordon, 2003) and compromise host defense system (McEwen, 1998). Clougherty et al. (2007) showed that traffic air pollution was associated with asthma among children exposed to violence, but not among children who are not exposed to community violence. Chen et al. (2008) reported that allergy related biological markers (IL-5, IgE, and eosinophil) increased by high chronic family stress only among low pollution exposure, but not among high low pollution exposure. These findings suggest that psychosocial stress, such as exposure to violence, changes the susceptibility of the impact of low-level air pollution on asthma. Clougherty et al. (2006) showed that fear of violence modified the efficacy of environmental improvement intervention targeting childhood asthma (e.g. replacing the child's mattress, industrial cleaning, integrated pest management [IPM], in-home education about IPM). They found that efficacy of the intervention had less impact of quality of life of children who feel high fear on violence than children who feel low fear on violence, suggesting that fear of violence might change the susceptibility of allergens.

However, the interaction pathway how “victimization” of violence associated with the susceptibility of air pollution on asthma was not reported. In addition, the association might be confounded by genetic factors, that is, those who are prone to feel fear on violence might share the genetic factors on allergic reaction. Future research needs to elaborate on how individual difference on personality, especially perception of violence, is associated with susceptibility of allergens genetically.

## Indirect Pathway of Violence on Asthma: Behavioral Change

The other indirect pathway how exposure to violence associates with asthma is the behavioral change. The plausible behavioral change due to exposure to violence would be 1) staying indoors, 2) skipping medication, and 3) smoking.

1. Staying indoors
  Parents in high-violence communities may restrict their children's outdoor activity (Wright and Steinbach, 2001; Levy et al. 2004) and keep indoors, where air pollutants are higher than outdoor in disadvantaged neighborhoods (Baxter et al. 2007; Spengler and Sexton, 1983). It has been reported that the behavior of keeping children indoor due to fear of violence is associated with increased risk of wheeze and physician's diagnosis of asthma (Wright et al. 1998b). Given that the deteriorated housing has been associated with increased cockroach allergen levels (Rauh et al. 2002), which is a known risk factor for increased asthma (Rosenstreich et al. 1997), children who live in disadvantaged neighborhoods and must stay indoors have higher rates of asthma morbidity (Wright and Steinbach, 2001). In addition, staying indoor would lead to obesity, and recent study found that obesity is associated with asthma (Stenius-Aarniala et al. 2000). Furthermore, keeping children at home would contribute to social isolation, which is another risk factor for asthma (Mailick et al. 1994).
2. Skipping medication
  Exposure to violence may change the compliance with therapy and medical follow-up for asthma. It is reported that violence could be a barrier to keeping appointments and following prescribed exercise program, due to fear of making a trip across town to a pharmacy or medical facility as a result of prior victimization or a perceived threat of violence (Fong, 1995). This may lead to misuse of prophylactic medication, delayed intervention, and consequently, greater likelihood of death. Additionally, pharmacies may not stay open at night in high crime areas, limiting immediate or emergency access to medication (Robicsek et al. 1993).
  In addition to skipping visits to medical facilities, exposure to violence might lead to a change in self-management through reducing perceived control (global feeling of the ability to deal with an environment). Research has demonstrated that populations face a greater deleterious effect of stress when facing daily life experiences that are unpredictable or uncontrollable (Cohen et al. 1995; Shagena et al. 1988; Holden, 1991). Lack of control would lead to low self efficacy, which may, in turn, be associated with poor asthma management, such as skipping self-medication for asthma (Clark et al. 1986).
3. Smoking
  It is known that smoking, another trigger of asthma, is a strategy used to cope with negative emotions or stress (Beckham et al. 1995; Acierno et al. 1996), and associated with deprivation of neighborhood (Kleinschmidt et al. 1995) and exposure to violence (Curry et al. 1993). In addition, lack of perceived control, helplessness, and low self-efficacy due to violence would contribute to smoking behavior as well (DuRant et al. 1995). Smoking due to exposure to violence might occur among mother during pregnancy (Lux et al. 2000), parents during infancy or young childhood (Strachan and Cook, 1998), or children themselves when they can initiate (Strine et al. 2004).

## Policy Implication

Asthma morbidity is the result of a complex interplay of influences operating at several levels, from the individual to the community, including environmental justice, human services, and law enforcement. However, prevention policy for asthma focusing on one of these factors alone won't suffice. For example, reinforcing police presence may not necessarily reduce the prevalence of psychosocial stress since police presence by itself may increase community stigma and fear. There is a need for intersectorial policies to simultaneously address exposure to violence as well as prevention and treatment of asthma morbidity. For example, fund to prevent asthma can be allocated to violence prevention, and future research on violence prevention should have asthma morbidity as outcome measurements.

One possible prevention policy would be to enhance social capital within the community to buffer the psychosocial stress associated with violence. Several researchers have found a link between low social capital and high crime or violence within community (Kennedy et al. 1998; Galea et al. 2002; Hemenway et al. 2001), and recent study showed the impact of community-level social capital on individual health, cancelling the effect of genetic and early family environment using twin study (Fujiwara and Kawachi, 2008). Social capital is defined as “those features of social structures, such as levels of interpersonal trust and norms of reciprocity and mutual aid, which act as resources for individuals and facilitate collective action” (Kawachi and Berkman, 2000). Residents living in a community with higher social capital tend to show better collective efficacy, which is associated with reduced fear or crime (Sampson et al. 1997). That is, in communities with better social capital, as social trust is high, people tend to collaborate with each other to prevent crime or violence. For example, “Neighborhood Watch” activity by community organization (Laycock and Tilley, 1995) might reduce the fear of violence in the community, prevent unsafe disposal of hazardous toxins, and in turn, prevent asthma.

## Conclusion

Exposure to community violence creates high levels of psychosocial stress in neighborhoods which are associated with a higher burden of childhood asthma. This review has demonstrated that there is sufficient evidence to argue that asthma is the embodiment of the exposure to the environmental pollutant of violence in children's lives, directly and indirectly.

There are many pathways through which this experience acts upon the body. Psychosocial stress due to violence directly influence on the development and/or exacerbation of asthma through biological responses, such as immune suppression and inflammation by dysregulation of HPA axis. The psychosocial stress might change the susceptibility of air pollution which has an impact on asthma. Exposure to violence would change the behavior of caretaker and/or children, such as keeping indoor, skipping medication, and smoking. These changes might induce greater exposure to indoor pollutants and allergens, sedentary lifestyle, and lack of social support, which are considered as risk of asthma, Based on this review, continued research and data gathering to elucidate the complex mechanism of the pathway how exposure to violence is associated with asthma is needed. Intersectoral collaboration, especially joint research between sectors where addressing violence and asthma is needed.

## Disclosure

The author reports no conflicts of interest.
